# Costs of hospital admissions due to COVID-19 in the federal capital of Brazil: a study based on hospital admission authorizations

**DOI:** 10.1016/j.bjid.2024.103744

**Published:** 2024-04-24

**Authors:** Ana Carolina Esteves da Silva Pereira, Luciana G. Gallo, Ana Flávia de M. Oliveira, Maria Regina F. de Oliveira, Henry M. Peixoto

**Affiliations:** a Universidade de Brasília, Núcleo de Medicina Tropical, Programa de Pós-Graduação em Medicina Tropical, Brasília, DF, Brazil; b Universidade de Brasília, Núcleo de Medicina Tropical, Zika, Arbovirus and other Infections Cohort Studies (ZARICS), Brasília, DF, Brazil; c Instituto Federal de Educação, Ciência e Tecnologia do Tocantins (IFTO), Tocantins, TO, Brazil; d Instituto de Avaliação de Tecnologia em Saúde (IATS), Porto Alegre, RS, Brazil

**Keywords:** COVID-19, Economic analysis, Hospital costs

## Abstract

This is a cost analysis study based on hospital admissions, conducted from the perspective of the Brazilian Unified Health System (SUS), carried out in a cohort of patients hospitalized at the University Hospital of Brasília (UHB) due to Severe Acute Respiratory Infections (SARI) caused by COVID-19, from April 1, 2020, to March 31, 2022. An approach based on macro-costing was used, considering the costs per patient identified in the Hospital Admission Authorizations (HAA). Were identified 1,015 HAA from 622 patients. The total cost of hospitalizations was R$ 2,875,867.18 for 2020 and 2021. Of this total, 86.41 % referred to hospital services and 13.59 % to professional services. The highest median cost per patient identified was for May 2020 (R$ 19,677.81 IQR [3,334.81–33,041.43]), while the lowest was in January 2021 (R$ 1,698.50 IQR [1,602.70–2,224.11]). The high cost of treating patients with COVID-19 resulted in a high economic burden of SARI due to COVID-19 for UHB and, consequently, for SUS.

## Introduction

Between December 2019, when the first cases of COVID-19 were detected, and April 2023, more than 760 million cases and 6.8 million deaths were recorded worldwide.^1^ In Brazil, the first case was confirmed in March 2020, and until April 2023, more than 37 million cases and 700,000 deaths were reported, of which 901,000 cases (2.4 %) and 11.8 thousand deaths (1.7 %) occurred in the Distrito Federal (Federal District - DF)^2^ – where 1.45 % of Brazilians reside.^3^^,^^4^

Given the ability to transmit the virus, the severity of clinical conditions, and several uncertainties regarding the pathophysiology and manifestations of the disease, health services needed to reorganize to serve the population quickly.^5^^,^^6^ In light of the available evidence, different strategies were created to expand care capacity and improve care for cases that required hospitalization, especially the most severe cases requiring hospitalization.^6^^,^^7^ This scenario resulted in the need to increase and relocate financial, professional, and even facility resources to face the imposed challenges.^5^^,^^8^ Studies of hospital costs of COVID-19 and measures taken to face the pandemic have been reported in different countries.^6^^,^^9^^,^^10^ However, there is still a shortage of information in the Brazilian context.

In general, individuals who need longer hospitalization require more resources for their care^11^ generating higher direct costs to the health system. Da Silva Etges et al. (2021) analyzed the investments made in 10 Brazilian hospitals, including public, public university, and private hospitals, to meet the high demand during the pandemic. They found that the total investment per patient ranged from USD 1,390 in a public hospital to USD 27,474 in a public university hospital.^5^ These values represent the investments allocated to COVID-19 hospitalized patients, covering expenses for the acquisition of equipment, infrastructure, and personal protective equipment.^5^^,^^12^, ^13^, ^14^

Existing literature reveals nuances related to the costs of hospitalization due to COVID-19. In Brazil, where healthcare is considered a right for all citizens that must be provided by the government, the challenge extends beyond acquiring material resources and infrastructure. Rocha et al. (2023) highlighted various variables that influence hospital costs, including Intensive Care Units (ICUs) bed services, consultations, medical procedures, medications, and supplies.^15^ Daily costs for ICU bed services and/or general medical ward and nursing services accounted for almost half of the total costs, followed by costs for medications and oxygen, which together accounted for 40 % of the total costs, demonstrating the extent of resources required to provide appropriate care to patient.^15^ Additionally, Santos et al. (2021), in a study that assessed public expenditures on hospitalizations, identified that patients requiring admission to ICUs incur considerably higher costs, with a 60.9 % increase compared to patients outside the ICU.^16^

There is a disparity in average costs per admission, depending on the perspective and region analyzed.^16^^,^^17^ This difference reflects different financing systems, management models, services, and resources available in different care settings. It suggests significant variations in resource utilization patterns and costs associated with disease management, pointing to differences in access to health care and technologies in different geographic and institutional contexts.^16^^,^^17^ Although there is a knowledge base on the economic burden of the disease at the national level in Brazil, localized investigations are still necessary to offer differentiated perspectives and inform specific strategies.

In this scenario, knowing the hospitalization costs for COVID-19 is important for understanding a considerable part of the economic burden of the disease and guiding the planning of investments in prevention and treatment.^10^ Thus, this study estimated hospitalization costs for Severe Acute Respiratory Infection (SARI) due to COVID-19 in adult patients at the University Hospital of Brasília (UHB), located in the Federal District of Brazil, between 2020 and 2021.

## Method

### Design

This is a cost analysis study from the perspective of SUS, based on the incidence of cases of SARI due to COVID-19 that required hospitalization at a UHB from April 1, 2020, to March 31, 2022, forming a patient cohort restricted to the hospitalization period. An approach based on macro-costing was used, considering the costs per patient identified in the Hospital Admission Authorizations (HAA).^18^ Hospital, professional, and total costs (hospital + professionals) were analyzed at two levels of aggregation identified in the HAA: by group (higher aggregation) and by procedure (lower aggregation). The procedures are aggregated into eight groups according to the activity area and purpose of the actions carried out, as follows: health promotion and prevention actions; procedures with diagnostic purposes; clinical procedures; surgical procedures; organ, tissue, and cell transplantation; medicines; orthoses, prostheses, and special materials; and complementary actions to healthcare.^18^

### Context of the study

In Brazil, the Unified Health System (SUS) is a public and free system that aims to ensure universal, integral, and equitable access to health for the entire population, ranging from basic care to complex procedures.^12^ In this context, the SUS Hospital Information System (Sistema de Informação Hospitalar ‒ SIH-SUS) is an administrative, electronic record-keeping tool that collects and organizes information about the costs of hospital care, the procedures performed, and other relevant data.^13^^,^^14^ The costs of the procedures performed by the SUS are standardized, and the value is passed on to these services listed in the health facilities’ records.^13^^,^^14^

### Hospital characterization

The hospital is strategically located in the downtown (central) area of the Distrito Federal and is characterized as a tertiary teaching hospital affiliated with SUS. The hospital mainly receives referral patients of medium and high complexity. However, there is spontaneous demand for emergency patients in the maternity ward, transplanted patients, and radiotherapy treatment.^19^ It offers specialized care in several areas, including chronic kidney disease and transplants. It is also a referral unit for monitoring high-risk pregnancies and the health of older adults. The hospital has 212 beds, 19 of which are in an adult Intensive Care Unit (ICU).^19^

In order to respond to the public health emergency of international concern caused by COVID-19,^20^ the hospital became part of the system regulated by the DF State Health Secretariat for COVID-19. Between 2020 and 2022, the institution provided up to 223 beds, of which 192 were regulated and of which 40 of the regulated beds (20%) were exclusive to patients hospitalized with COVID-19 (20 ICU beds and 20 ward beds).^19^

### Participants

All hospitalized patients with a positive laboratory test for COVID-19 and SARI were considered eligible. Patients with negative or suspected tests without laboratory confirmation were not included.

**Fig. 1:**
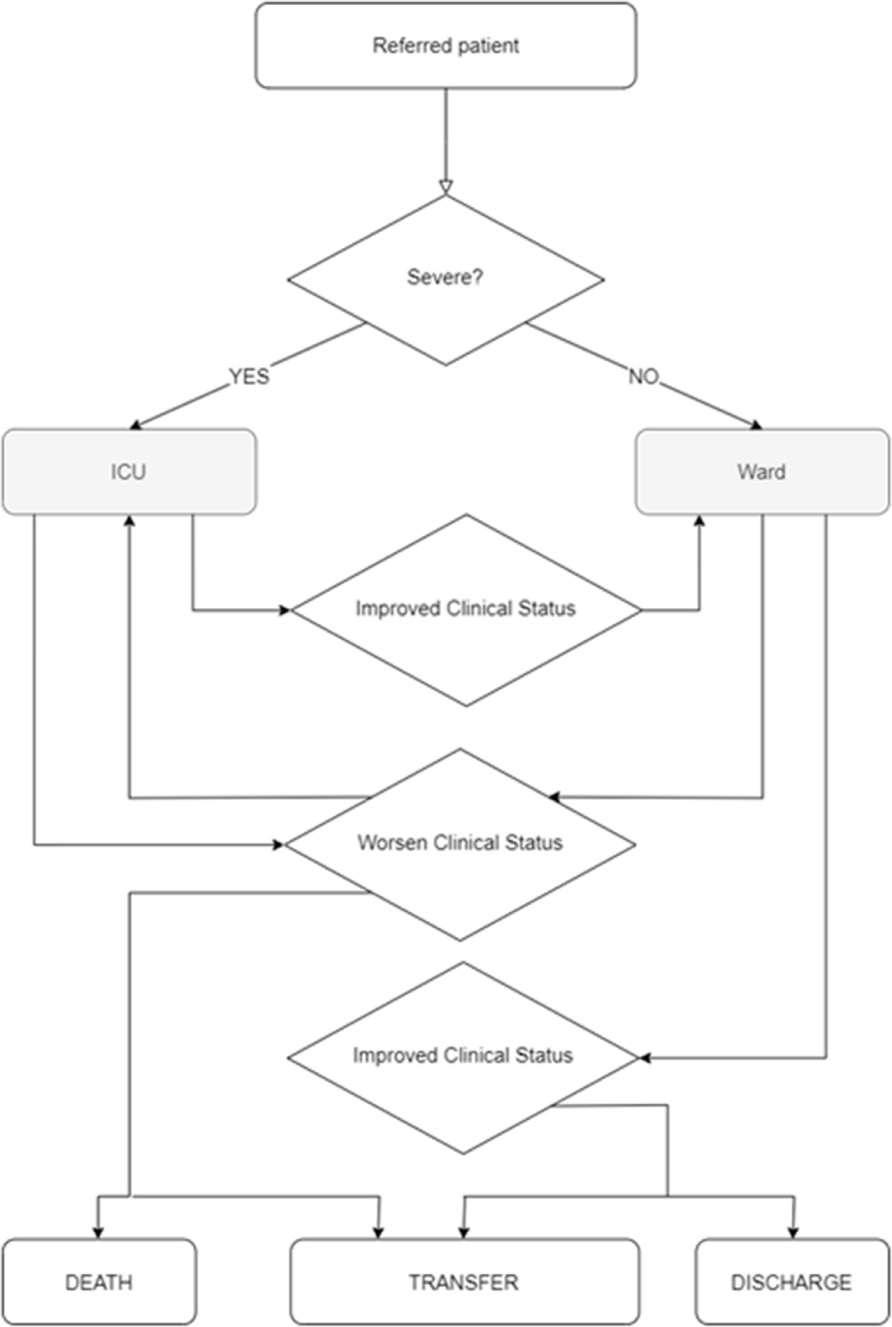
Flow of patients admitted to the hospital.

Based on clinical severity, patients referred to the hospital were directed to the ICU or the ward. Those in the ICU whose clinical status had improved were transferred to the ward; those cases of worsening clinical condition remained in the ICU, with the possibility of death or transfer to another health service. Patients in ward beds with worsening clinical status were transferred to the ICU, and if there was improvement were transferred to other health services or were discharged (Fig. 1).

To conduct the study, patients were identified based on Hospital Epidemiological Surveillance records (records of suspected/confirmed cases of COVID-19). In laboratory diagnostic confirmation, patients who tested positive through Reverse Transcription Polymerase Chain Reaction (RT-PCR) and/or Immunoglobulin M (IgM) serology were considered eligible, as recorded in electronic medical records. Regarding the presence of SARI, two medical professionals, hospital workers, and research team members independently evaluated the medical records, and the uncertainties were resolved by consensus.

From the identification of eligible patients, (i) The identifier of the hospital medical record and (ii) The period of hospitalization ‒ between the start date of hospitalization for COVID-19 and the date of hospital discharge (exit for any reason) ‒ were collected in the electronic medical record. With these data, the HAA were identified in the records made available by the hospital's billing department. All HAA generated during the hospitalization period of eligible patients were considered, including those whose main reason for hospitalization was something other than COVID-19.

### Data sources and variables

The HAA is a SIH instrument developed and used for payments to health facilities financed by the SUS. Therefore, the procedures performed during hospitalizations are recorded in the HAA. It has more than 50 variables related to hospitalization, from which the following were collected: HAA number, medical record identifier, date of hospitalization, date of departure, reason for departure, date of birth, sex, race, municipality of residence, Federative Unit/State (FU) of residence, zip code, main International Classification of Diseases (ICD), main and secondary procedures, number of procedures, values per procedure and type of service (hospital and professional).

The procedures are organized by a code built according to their characteristics. There are eight different groups divided into subgroups and forms of organization, as the procedures are more specific. The main procedure, that is, the larger one, and the secondary ones were used for analysis. Each procedure has a tabulated value comprising professional and/or hospital services.

During hospitalization, more than one HAA can be generated per patient. Thus, for this study, all HAA generated during the hospitalization period due to COVID-19 were considered, and none of the procedures contained in the HAA were excluded. As for the outcome, the last record was considered for patients with more than one HAA during the hospitalization period.

### Data analysis

All data were extracted from the HAA records, tabulated, and analyzed in Microsoft Excel® (version 2016). The sociodemographic and clinical data of the patients were presented descriptively, and measures of central tendency and dispersion were estimated for numerical variables and absolute and relative frequencies for categorical variables.

The Shapiro-Wilk test was used in the Stata program (version 17) to test the normality of data on costs, age, amount of HAA per patient, number of days, and procedures. The median and interquartile ranges were used to present cost results, considering that the data did not present a normal distribution. The other numerical variables presented a normal distribution; therefore, the means and corresponding standard deviations were presented.

For analyses by health region, the division defined by the State Department of Health of the Distrito Federal was used to categorize the patient's place of residence. The zip code registered in the HAA was used to identify the location. The costs were presented in Brazilian currency (reais, R$). To facilitate comparison with other currencies, it is recommended to convert them into American dollars (USD) using the exchange rate from March 31, 2022 (R$4.74 per dollar).

### Research ethics

The study protocol was approved by the Research Ethics Committee of the Faculty of Medicine of the University of Brasília, opinion n° 4.112.214, and the use of a free and Informed Consent Form (ICF) was waived due to the exclusive use of secondary administrative data to carry out this study. The subjects who made up the study sample were selected by hospital surveillance professionals who preserved the sensitive data. Care was taken to avoid exposing the research subjects at all stages of the study.

## Results

Of the 622 eligible patients, 1,015 HAA were identified, with a total of 10,902 days of hospitalization, of which 806 (7.39 %) corresponded to ICU admissions. Between April 4, 2020, the day of the first hospitalization, and December 31, 2020, 232 patients were hospitalized; from 2021 until December 1, 2021, the date of the last hospitalization, 390 patients were hospitalized. Of the total patients, 58.43 % were male, with a mean age of 61 years (SD ±16.19), and 11.34 % of these men were older than 80. As for race/skin color, 280 patients had no information available; among those with available information, 191 (55.85 %) were black/brown, and 92 (26.90 %) were white. Regarding the place of residence, 94.69 % were residents of the Distrito Federal or neighboring municipalities that are part of the SUS network referral service. On average, 58 professional procedures were recorded per patient. It was not possible to identify the final outcome of 61 (9.81 %) patients, as there was a transfer, administrative closure, or change of procedure, and 167 (26.85 %) died (Table 1), corresponding to 29.77 % of those with information about the outcome. Only 75 (12.06 %) patients were admitted to the ICU, totaling 806 days of hospitalization; of these patients, 51 were hospitalized in 2020, totaling 584 (72.46 %) day of hospitalization.

**Table 1:** Distribution of patients hospitalized with SARI* due to COVID-19 in UHB⁎⁎ according to sociodemographic characteristics, 2020–2022^a^.

Characteristics	*n*	%	Average	SD
Sex and Age Range				
Male	362	58.43	61.22	16.39
0 to 19	‒	‒	‒	‒
20 to 29	9	0.57	‒	‒
30 to 39	31	2.83	‒	‒
40 to 49	46	5.40	‒	‒
50 to 59	73	10.55	‒	‒
60 to 69	85	14.38	‒	‒
70 to 79	68	13.35	‒	‒
80 and more	50	11.34	‒	‒
Female	260	41.57	60.64	15.90
0 to 19	1	0.05	‒	‒
20 to 29	10	0.70	‒	‒
30 to 39	14	1.33	‒	‒
40 to 49	37	4.37	‒	‒
50 to 59	56	8.00	‒	‒
60 to 69	60	10.11	‒	‒
70 to 79	47	9.13	‒	‒
80 and more	35	7.87	‒	‒
**Age**	622	‒	60.97	16.19
**Race/Color**				
Black/Brown	191	30.71	‒	‒
White	92	14.79	‒	‒
Yellow	57	9.16	‒	‒
Indigenous	2	0.32	‒	‒
No information	280	‒	‒	‒
**DF Health Region**				
Central	41	6.59	‒	‒
Central-southern	64	10.29	‒	‒
Eastern	55	8.84	‒	‒
Northern	76	12.22	‒	‒
Western	96	15.43	‒	‒
South-western	136	21.86	‒	‒
Southern	53	8.52	‒	‒
Surroundings	68	10.93	‒	‒
Other federal units	32	5.14	‒	‒
No information	1	0.16	‒	‒
**HAA**	1015	‒	1.63	1.14
**Procedures**				
Hospital Service	24,415	40.17	39.25	63.27
Professional service	36,362	59.83	58.46	69.80
Days of hospitalization				
Total inpatient days	10,096	92.61	16.23^b^	17.00
ICU days	806	7.39	1.30^b^	5.14
**Outcome**				
Discharges	394	63.34	‒	‒
Death	167	26.85	‒	‒
Transfer	52	8.36	‒	‒
Change of procedure/administrative closure^c^	9	1.45	‒	‒
**Main ICD-10**			‒	‒
Coronavirus infection of unspecified location	485	77.97	‒	‒
Other ICD-10	137	22.03	‒	‒

a One patient was hospitalized in 2021 with an outcome on January 27, 2022.

b Average number of days of hospitalization per patient.

c Procedure change occurs due to complications that involve changes in conduct or medical specialty; Administrative closure is used when it is necessary to issue new HAA to the same patient during admissions.

⁎ SARI, Severe Acute Respiratory Infection.

⁎⁎ UHB, University Hospital of Brasília.

**Fig. 2:**
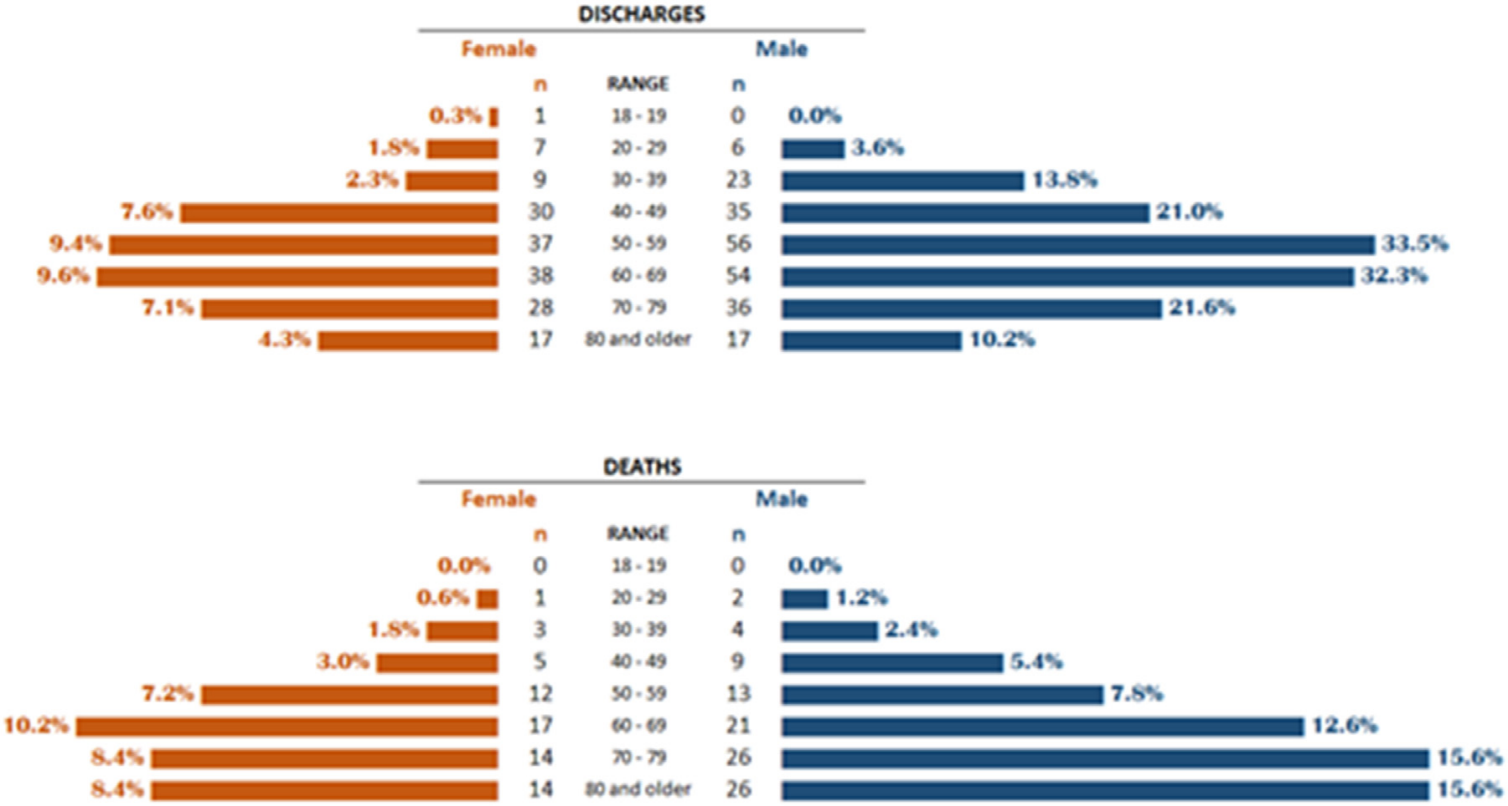
Distribution of SARI* patients due to COVID-19 according to outcomes of discharges and deaths by age group and sex. UHB**, 2020 and 2021. *SARI: Severe Acute Respiratory Infection; **UHB, University Hospital of Brasília.

Of the 622 patients, 232 (37.3 %) were hospitalized in 2020 and 390 (62.7 %) in 2021. Approximately 39 % of hospitalizations in 2020 and 20 % in 2021 had death as an outcome, while for the discharge outcome, these percentages were approximately 53 % and 69 %, respectively. Observing discharges and deaths alone, among discharges, there was a higher frequency between the age range of 50 to 69 (46.95 %) for both sexes and death in women from age 60 (26.95 %) and in men from age 50 (51.50 %) (Fig. 2).

**Fig. 3:**
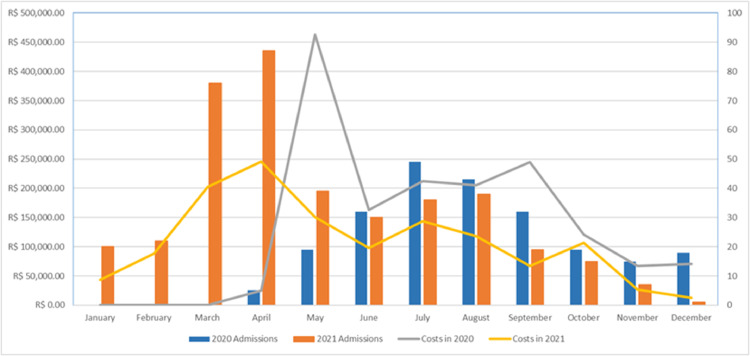
Monthly distribution of costs and total hospitalizations of patients with SARI* due to COVID-19 in UHB**, 2020 and 2021. *SARI, Severe Acute Respiratory Infection; **UHB, University Hospital of Brasília.

The total cost for hospitalizations was R$2,875,867.18 in 2020 and 2021, ranging from R$462,745.55 in May 2020 to R$12,836.16 in December 2021 (Fig. 3). Of this total, R$2,485,071.99 (86.41 %) referred to hospital services and R$390,795.19 to professional services (13.59 %). The median cost per patient for May 2020 was the highest median cost identified (R$19,677.81 IQR [3,334.81–33,041.43]), while the lowest was in January 2021 (R$1,698.50 IQR [1,602.70–2,224.11]). The individual value of hospitalization per patient ranged from R$129,228.14 for patients hospitalized in May 2020 to R$215.33 for patients hospitalized in October 2020.

**Fig. 4:**
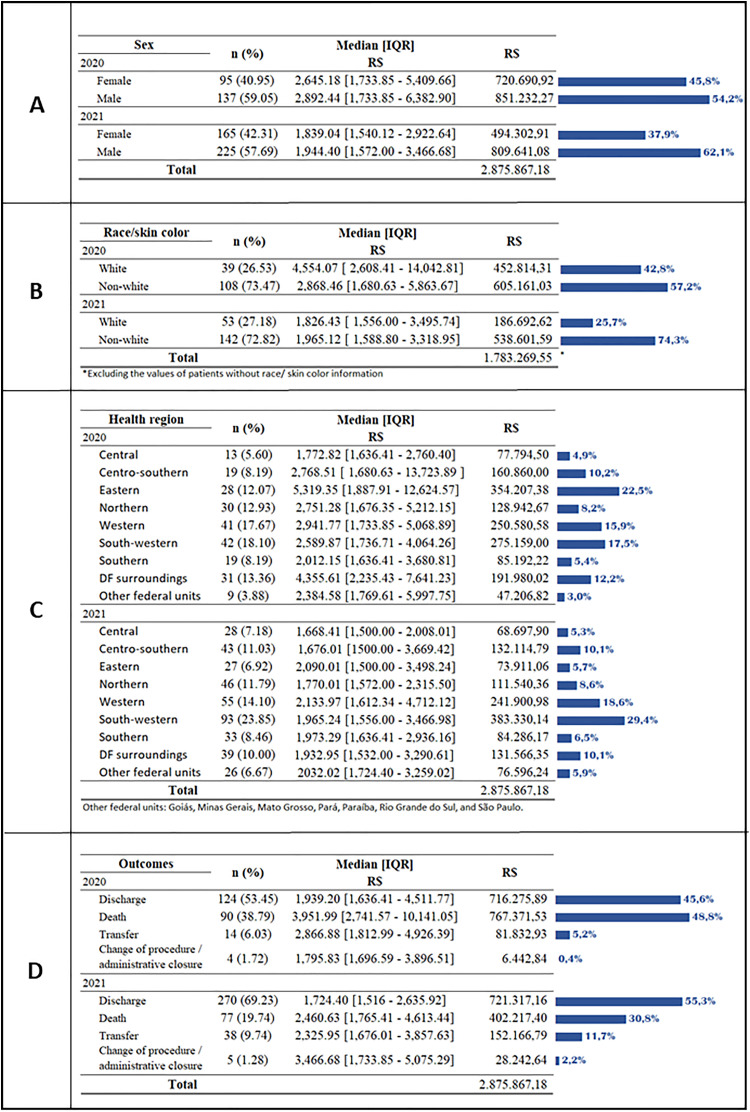
Distribution of costs of patients hospitalized with SARI* due to COVID-19 in the UHB** according to sociodemographic characteristics, 2020 and 2021: (A) Sex; (B) Race/color; (C) Health region; (D) Outcomes. *SARI, Severe Acute Respiratory Infection; **UHB, University Public Hospital.

The median costs in 2020 were higher for men (R$2,892.44 IQR [1,733.85–6,382.92]) (Fig. 4A) and white people (R$4,554.07 IQR [2,608.41–14,042.81]) (Fig. 4B); however, in 2021, non-white people presented a higher median value (R$1,965.12 IQR [1,588.80–3,318.95]). Patients living in the eastern health region of the DF represented 12.07 % of hospitalized patients in 2020 and 22.5 % of costs for this year. In 2021, patients living in the southwest region accounted for 23.85 % of hospitalized patients and 29.4 % of costs (Fig. 4C). In both years, the southwest was the health region with the highest number of hospitalization records. In 2020, the 90 patients (38.79 %) with a death outcome were responsible for the largest share of costs, R$767,371.53 (48.8 %) (Fig. 4D).

Among patients hospitalized in 2021, nine were transferred from Manaus, northern Brazil, due to a lack of oxygen and overcrowding of health services. These patients totaled R$15,069.10 (1.16 %) of the costs for 2021, 66 day of hospitalization in the ward; six (66.67 %) were discharged, one (11.11 %) died, and two (22.22 %) were transferred to another establishment.

Of the 60,777 procedures, 36,227 (59.61 %) were performed in 2021. In 2020, complementary healthcare actions, the group in which per diem rates are recorded, were responsible for most of the costs: R$826,325.12 (53.64 %), followed by clinical procedures, at R$503,344.33 (32.67 %). In 2021, clinical procedures represented the largest share of costs: R$751,854.86 (56.34 %), followed by surgical procedures at R$249,086.61 (18.67 %) **(**Table 2).

**Table 2:** Distribution of costs according to groups of procedure and years of patients hospitalized with SARI* due to COVID-19 in the UHB⁎⁎, 2020 and 2021.

Procedures group^a^	*n*	Hospital service costs (R$)	Professional service costs (R$)	Total costs (R$)	%	Median [IQR] (R$)
**2020**						
Procedures with diagnostic purpose	793	68,818.49	‒	68,818.49	4.47 %	136.41 [47.52‒138.63]
Clinical procedures	17,105	411,985.58	91,358.75	503,344.33	32.67 %	46.77 [15.00‒116.92]
Surgical procedures	3389	133,163.10	8654.26	141,817.36	9.21 %	212.77 [78.00‒712.80]
Organ, tissue and cell transplantation	0	0	0	0	‒	‒
Medicines	40	24.00	‒	24.00	0.00 %	12.00 [4.20‒19.80]
Orthoses, prostheses, and special materials	13	204.64	‒	204.64	0.01 %	8.92 [8.93‒8.93]
Complementary healthcare actions	3,210	712,320.82	114,004.30	826,325.12	53.64 %	144.00 [48.57‒1372.80]
Total in 2020	24,550	1,326,516.63	214,017.31	1,540,533.94	100 %	70.00 [22.54–165.24]
2021						
Procedures with diagnostic purpose	1,174	94,161.71	85.99	94,247.70	7.06 %	116.92 [39.94‒138.63]
Clinical procedures	23,287	626,593.37	12,521.49	751,854.86	56.34 %	49.04 [15.00‒195.38]
Surgical procedures	5,506	229,971.77	19,114.84	249,086.61	18.67 %	196.66 [78.00‒562.64]
Organ, tissue and cell transplantation	78	42,147.99	16,053.84	58,201.83	4.36 %	382.62 [76.81‒2487.00]
Medicines	278	1,534.62	‒	1,534.62	0.12 %	213.12 [18.00‒264.00]
Orthoses, prostheses, and special materials	31	10,143.95	‒	10,143.95	0.76 %	97.48 [8.93‒237.02]
Complementary healthcare actions	5,873	153,295.38	16,072.78	169,368.16	12.69 %	80.00 [38.85‒144.00]
Total in 2021	36,227	1,157,848.79	63,848.94	1,334,437.73	100 %	71.53 [23.38–194.40]
Overall total	60,777	2,484,365.42	277,866.25	2,874,971.67		**70.00 [23.00**‒**182.40]**

a The groups of procedures are divided into (i) Procedures with diagnostic purposes: imaging, pathological, cytopathological diagnostics, rapid tests, and collection of material, among others; (ii) Clinical procedures: appointments, consultations, physiotherapies, treatments in general, labor and delivery; (iii) Surgical procedures: surgeries in their various specialties, minor surgeries, dressings, drainages, debridement, accesses for dialysis, among others; (iv) Organ, tissue and cell transplantation: collection of tests for donation purposes, assessments of brain death, transplantation, and pre- and post-transplant follow-up; (v) Medicines: exceptional, strategic, hospital and urgent and specialized medicines for pharmaceutical care; (vi) Orthoses, prostheses, and special materials: inputs related or not to surgery, and (vii) Complementary healthcare actions: per diem rates, authorizations, and regulations. The procedure costs cover most of the expenses for necessary supplies and medications.

⁎ SARI: Severe Acute Respiratory Infection.

⁎⁎ UHB, University Hospital of Brasília.

Of the total cost of clinical procedures performed (R$1,255,199.19), R$661,645.07 (52.71 %) were directed to treatment of COVID-19, of which R$382,631.28 was in 2021, and R$193,908.40 (15.45 %) for hemodialysis, of which R$137,467.71 was in 2021. Among the costs of surgical procedures, 79.46 % (R$310,725.82) were due to pressure wound care (Appendix A).

## Discussion

The total cost for 622 patients hospitalized with SARI due to COVID-19 in a university hospital in the Distrito Federal between 2020 and 2021 was R$2,875,867.18 ‒ with median cost R$2,094.53 IQR (1,636.41–4,009.66). May 2020 represented the highest median cost per patient, and January 2021 presented the lowest. In both years, male patients accounted for a greater share of total costs. As for the outcomes, in 2020, care for patients who died was costlier than for those who were discharged – although they represented the smallest share of hospitalized patients.

In relation to the 60,777 procedures recorded in the HAA during the total study period, clinical procedures made up the majority (43.66 % of the total procedures, 66.46 % of the total cost), and pressure wound care accounted for more than 79 % of the costs related to surgical procedures.

The negative effects of the COVID-19 pandemic concerning the high amount of limited-time deaths, social isolation, and loss of jobs, among other consequences, were experienced in all countries, with an even stronger economic and social impact in developing countries.^8^^,^^21^ In 2020, hospital costs represented 55 % of total costs, despite the lower number of hospitalized patients (37 % of the total). This result may be associated with initial unawareness of the disease and the need to rapidly reorganize health services to ensure appropriate care for cases. This fact is reflected in hospital costs, given the increased demand for hospital services, the need to implement additional security measures, the reorganization of care flows, and the need to open new beds for patient care. When analyzing the years separately, it appears that the casuistry identified in the UHB in 2020 is in line with the findings of Carvalho et al.^22^ who pointed to a significant increase in the number of cases, ICU hospitalizations, and SARI deaths during the first year of the COVID-19 pandemic in Brazil. Therefore, the epidemiological context indicates the severity of the public health emergency we experienced in Brazil,^17^^,^^23^, ^24^, ^25^ especially in the first year, which may partially justify the higher costs identified in the present study for 2020.

The present study shows higher costs in 2020, which should be analyzed in the light of existing literature on the costs associated with COVID-19 in Brazil. A detailed examination of these costs reveals a complex scenario influenced by a number of factors, including pandemic containment measures and the pressure on the health care system due to additional demand.^15^^,^^16^^,^^26^ In the Brazilian context, Santos et al. (2021) revealed that the year 2020 had a significant economic impact, including additional costs for medical treatment, personal protective equipment, and hospital infrastructure. The total costs identified exceeded R$2 billion nationwide, with a sum of 37.3 million solley for the Federal District. The individual costs identified were over R$3,000 per hospitalization, which is slightly above to the results obtained in our study, with a median cost per hospitalization of R$2,094 IQR (1,636.41‒4,009.66).^16^

It is important to consider the beginning of the vaccination campaign on January 17, 2021.^27^ Although it occurred at a slow pace, with just over 11 % of the population of the Distrito Federal vaccinated by May 2, 2021, this factor may have been key to the reduction of costs observed from May 2021.^28^ The second wave of COVID-19 cases in Brazil also deserves special attention since it has resulted in a health crisis characterized by a shortage of hospital beds for patients with COVID-19 in some Brazilian regions from January to April 2021.^29^, ^30^, ^31^ During this period, in addition to the increase in local demand, the transfer of patients between hospitals and Brazilian Federative Units may have entailed higher costs in the care of hospitalized patients in the UHB.

The gradual reduction of costs per case throughout 2021 may have resulted from the positive impact of vaccination, which contributed to the decrease in the number of reported and severe cases. In addition, there was an improvement in the quality of care provided to hospitalized patients due to the knowledge acquired.^32^, ^33^, ^34^

The present study identified a higher cost per male patient, as well as for patients living in the eastern health region of the Distrito Federal and patients living in other federal units. These results may have been caused by factors such as the resistance of Brazilian men to seek healthcare^35^ the greater social and economic vulnerability identified in the eastern region,^36^ and the challenges related to access to healthcare faced by inhabitants of other federal units located in the DF area.^37^ It is plausible to assume that these patients were referred for hospitalization belatedly when the disease was already more severe, which resulted in more costly care. In addition, it is important to note that vulnerable communities have a higher proportion of uncontrolled comorbidities, which is associated with greater disease severity.^38^, ^39^, ^40^

With regard to patients transferred from Manaus, despite coming from a locality that at the time faced an intense health crisis, with overcrowding of hospitals and lack of oxygen,^30^ they generally had short periods of hospitalization, low costs, and favorable outcomes. Such characteristics may have occurred due to the need for the patient to be transferred when in a stable clinical condition. In addition, the present analyses did not include the costs of the procedures carried out in the hospital of origin and the costs of interstate transfers.

The present study was conducted at a university hospital recognized for its quality of care and reference to various conditions requiring excellence in care. The high cost related to pressure wound care may reflect the clinical conditions in which patients were admitted, structural challenges, such as worker overload due to the high demand of patients, in addition to the clinical instability of the patients and their health condition.^41^^,^^42^ COVID-19 can cause muscle weakness, loss of function, and limitations in mobility, and in severe cases requiring mechanical ventilation, hemodynamic instability, and other medical devices can limit the ability to move and reposition the patient.^43^ It is also noteworthy that some patients hospitalized with COVID-19 often have other pre-existing health conditions, such as diabetes, cardiovascular diseases, and immunosuppression, which can compromise skin healing and increase the risk of developing pressure wounds.^43^

Among the limitations of this study, we highlight the use of HAA as a data source. Although HAA reflect the value passed on by SUS to the hospital, they may not reflect all procedures performed during the hospitalization period.^44^ Thus, it is possible that not all procedures performed have been recorded and that some procedures, particularly procedures of highly complex nature, do not make up the coverage of the HAA.^44^^,^^45^ It is important to note that in the HAA, procedure costs cover most of the expenses of supplies and medications required to perform the procedure, except for some that are billed separately due to their specific characteristics. Therefore, the listed values for the procedures reflect not only the cost of the procedure itself but also the costs associated with the supplies and medications used during the process.^46^ Besides, it is known that the real cost of some procedures may be higher than that passed on by SUS, with a co-investment of the hospital in patient care.^45^ In this regard, the real costs may be underestimated, especially considering the scenario of emerging conditions and health crises experienced while the study was being carried out. Moreover, this study did not aim to estimate the total costs from the perspective of SUS and thus did not include other factors, such as costs related to patient transportation, outpatient care, and hospitalizations in other hospitals.

It should be noted that the study is unprecedented in the DF and was conducted from a cohort of patients; therefore, in addition to ensuring the patients included had, in fact, a confirmed diagnosis of COVID-19, it was possible to capture all HAA per patient in the period, even with the record of the main procedure not focused on COVID-19. Thus, it was possible to detail the different procedures and groups of procedures performed and the behavior of total and median costs throughout the hospitalization period.

## Conclusion

The results showed a higher cost for hospitalized patients in the first year of the pandemic in men and regions with greater social and economic vulnerability. Clinical procedures that include treatment for COVID-19 were the costliest during the analysis period.

It is known that macro-costing of diseases based on HAA may underestimate the real cost. Still, the high cost of treating patients with COVID-19 demonstrated a high economic burden of SARI due to COVID-19 for UHB and, consequently, for SUS.

## Funding

This work was supported by the Ministério da Educação (MEC)/Universidade de Brasília (UnB)/Faculdade de Medicina (FM) ‒ Integrated research and service actions to face the COVID-19 pandemic in the Distrito Federal, and by National Institute of Science and Technology for Health Technology Assessment (Instituto de Avaliação de Tecnologias em Saúde – IATS)

## CRediT authorship contribution statement

**Ana Carolina Esteves da Silva Pereira:** Conceptualization, Methodology, Formal analysis, Data curation, Investigation, Writing – original draft. **Luciana G. Gallo:** Conceptualization, Methodology, Investigation, Writing – original draft. **Ana Flávia de M. Oliveira:** Conceptualization, Methodology, Investigation, Writing – review & editing. **Maria Regina F. de Oliveira:** Conceptualization, Methodology, Writing – review & editing, Project administration. **Henry M. Peixoto:** Conceptualization, Methodology, Writing – review & editing, Project administration.

## Conflicts of interest

The authors declare no conflicts of interest.

## Appendix Supplementary materials

Image, application 1
