# Supplementary material for: Costs of hospital admissions due to COVID-19 in the federal capital of Brazil: a study based on hospital admission authorizations
Source: Braz J Infect Dis. 2024 Apr 24;28(2):103744. doi: 10.1016/j.bjid.2024.103744 (PMC11099317; doi:10.1016/j.bjid.2024.103744)
Supplement: Supplementary file 1 [file mmc1.docx]

**BJID-D-23-00312_Supplementary Material**

**Appendix A** Distribution of costs according to the form of organization of the procedure groups and years of patients hospitalized with SARI* due to COVID-19 in the UHB*, 2020 and 2021.

| Form of organization of procedures | n | R$ | % | Median [IQR] R$ |
| --- | --- | --- | --- | --- |
| **Clinical procedures** |  |  |  |  |
| Consultation/evaluation in hospitalized patient | 33843 | R$176,249.30 | 14.04 | 31.44 [10.13‒93.08] |
| Physiotherapeutic services | 2587 | R$15,778.80 | 1.26 | 76.20 [31.75‒140.10] |
| Hemodialysis for acute/acute chronic patients/dialysis initiated | 687 | R$193,908.40 | 15.45 | 955.47 [530.82‒2123.28] |
| Treatment of coronavirus infection ‒ COVID-19 | 1058 | R$661,645.07 | 52.71 | 750.00 [46.77‒1192.99] |
| Treatment of other diseases | 998 | R$154,306.71 | 12.29 | 80.07 [10.43‒333.06] |
| Other proceedings | 1219 | R$53,310.92 | 4.25 | 18.00 [15.00‒70.00] |
| **Total clinical procedures** | **40392** | **R$1,255,199.20** | 100.00 | 48.64 [15.00‒152.01] |
| **Complementary healthcare actions** |  |  |  |  |
| Per diem adult escort/Long stay per diem | 4304 | R$52,216.66 | 5.24 | 76.80 [38.40‒129.52] |
| Per diem pregnant companion | 35 | R$ 320.00 | 0.03 | 56.00 [24.00‒240.00] |
| Per diem older adult escort | 3131 | R$27,100.80 | 2.72 | 60.80 [28.80‒105.60] |
| Adult ICU per diem Rate | 690 | R$173,583.82 | 17.43 | 821.84 [406.80‒2465.52] |
| Adult ICU per diem rates ‒ COVID-19 | 922 | R$742,400.00 | 74.56 | 2745.60 [1363.20‒8236.80] |
| Cardiopulmonary bypass monitoring | 1 | R$72.00 | 0.01 | ‒ |
| **Total complementary healthcare actions** | **9083** | **R$995,693.28** | 100.00 | 88.00 [40.00‒225.33] |
| **Surgical procedures** |  |  |  |  |
| Anesthesia | 3 | R$399.00 | 0.10% | 100.80 [96.60‒201.60] |
| Surgeries of the upper airways (face of head and neck) | 9 | R$1,475.96 | 0.38% | 72.63 [58.00‒183.64] |
| Endocrine gland surgery | 3 | R$393.92 | 0.10% | 71.80 [55.00‒267.12] |
| Circulatory system surgeries | 20 | R$10,934.11 | 2.80% | 167.04 [53.01‒773.31] |
| Abdominal surgery | 47 | R$9,664.61 | 2.47% | 50.00 [28.30‒321.68] |
| Genitourinary system surgery | 28 | R$4,606.25 | 1.18% | 64.87 [45.02‒250.59] |
| Musculoskeletal system surgery | 6 | R$1,070.90 | 0.27% | 119.71 [35.00‒166.60] |
| Dialysis access | 71 | R$12,586.52 | 3.22% | 163.89 [163.89‒196.66] |
| Oncology surgery | 9 | R$7,968.42 | 2.04% | 326.95 [218.00‒1397.41] |
| Obstetric surgery | 2 | R$807.69 | 0.21% | 403.84 [146.90‒660.79] |
| Thoracic surgery | 211 | R$30,335.57 | 7.76% | 98.40 [50.40‒183.76] |
| Debridement of necrotizing/ulcerative/devitalized tissue fasciitis | 141 | R$24,247.26 | 6.20% | 78.00 [67.57‒327.17] |
| Grade II dressing with or without debridement/incision/abscess drainage | 8344 | R$268,413.80 | 73.27% | 486.00 [194.40‒972.00] |
| **Total surgical procedures** | **8894** | **R$372,904.01** | **100%** | 196.66 [78.00‒604.92] |

*SARI, Severe Acute Respiratory Infection; **UHB, University Hospital of Brasília.
